# The Future of Virtual Reality Therapy for Phobias: Beyond Simple Exposures

**DOI:** 10.32872/cpe.v2i2.2913

**Published:** 2020-06-30

**Authors:** Alexander Miloff, Philip Lindner, Per Carlbring

**Affiliations:** a Department of Psychology, Stockholm University, Stockholm, Sweden; b Centre for Psychiatry Research, Department of Clinical Neuroscience, Karolinska Institutet & Stockholm Health Care Services, Stockholm County, Stockholm, Sweden

Inelegant as they may look to the outsider, the white boxy Samsung Gear VR goggles with a smartphone strapped to the front, have the power to change lives. In the last few years our research team at Stockholm University have used the device to treat nearly 100 spider phobic patients with virtual reality exposure therapy (VRET) using the Itsy application, developed alongside VR-startup Mimerse (Miloff et al., 2016). The real tears patients shed may be indication enough that the animated spiders and computer-generated world are helping them face their deepest fears. However, evidence shows large reductions in self-reported fear and avoidance around live spiders. In fact, the positive behavior change is very nearly as powerful as the gold-standard treatment for spider phobia that ends with handling a 3-centimeter spider with their hands (Miloff et al., 2019). The boundary for how we perceive real and artificial may not be as large as we think.

Today, the biggest tech companies are still pouring enormous resources into making virtual a reality. Facebook purchased Oculus, shipped the Rift, the mobile Go and now Quest, Google had the Daydream-standard and is now moving onto augmented-reality, Sony the Playstation VR and even Apple is said to be working on a device. Still, there is a feeling in this industry that it isn’t really clear what virtual reality is good for. There are entertaining games available sure, mostly shooters and rhythm games. There is the extremely enjoyable feeling of awe to be dropped into a virtual world somewhere, flying in a fighter jet or swimming with divers. New ways of storytelling are certainly possible and are being created. However, there is the persistent feeling that something is missing. The technology is just too powerful for the limited experiences we’ve developed so far.

To understand what is possible, it may be best to look at the way our reality generating system functions and work backwards. Our eyes, ears, taste and touch are geared towards favoring certain information over others (Bayle et al., 2009; Erlich et al., 2013; Öhman & Mineka, 2001). Sudden movement in the corner of our eye evokes a fear response, as does the sound of a potentially violent individual above the din of a crowd, or the unexpected irritation of a wriggling bug on our skin. See a certain shape walk by and lust towards an attractive mate might cause butterflies in the stomach. The most common use of virtual reality in clinical treatments is for phobias and similar to face-to-face treatment is almost always seen through the lens of stimulus-emotion pairs and exposure therapy (Turner & Casey, 2014). With virtual reality, however, we might be able to explore not only working to modify basic emotions using simple stimuli but higher order functions of the mind using complex simulations as well.

For millions of years we sat on the savannah around open fires. The rustling and movement in tall grasses at the far edge of the camp may be just the wind but our minds see a leaping lion ready to disembowel us. Gifted with large brains capable of complex pattern recognition and learning, we’ve developed immense capabilities of prediction. For want of a better word, this is the power of imagination and at its most vivid. We see in our mind’s eye a disaster before we experience it. We feel ourselves drowning before we ever get on the boat. We feel the wind on our face and the sensation of hitting the ground before we ever step onto the airplane. In the right frame of mind, we may have even pictured the previous two sentences in our imagination as we read them. Although this capacity is one of the ways we define ourselves as human, it’s also responsible for great suffering, catastrophic fears, debilitating anxiety; its moderation actually one of the ways we define treatment success in specific phobia, i.e., no longer believing your catastrophic fears (Davis et al., 2012).

We are just at the beginning of exploring the many uses of VR and its practical application to clinical psychology. Tremendous progress has been made at importing what we know from traditional formats for psychological treatments (e.g., exposure therapy), but new and more innovative leaps in understanding and technique are possible. The capacity of imagination is something we take for granted and generalized solutions for dealing with its problematic aspects limited. Virtual reality offers a nearly limitless world in which to create, restricted only by development costs and again, the more useful aspects of our imagination. The industry driving development of the technology is searching for the killer app that could convince new users to jump in, and clinical applications that converge with the gaming industry and storytelling might offer such an opportunity. Whether such generalized solutions are possible is uncertain, however what is certain is that the future of clinical treatment and virtual reality is more than just simple exposures.
